# The Flipped Classroom Approach in a Pediatric Anesthesiology Fellowship Curriculum

**DOI:** 10.7759/cureus.43979

**Published:** 2023-08-23

**Authors:** Rachel Moquin, Megan Dewey, Ashley Weinhold, Ottavia Green, Anna Rebecca Young

**Affiliations:** 1 Department of Anesthesiology, Washington University School of Medicine, St. Louis, USA

**Keywords:** flipped classroom, adult learning theory, wellness and resilience, graduate medical education (gme), covid-19, fellowship training, curriculum development and evaluation, pediatric anesthesiology

## Abstract

Background

The flipped classroom approach is well documented to enhance medical education outcomes. Additionally, both the need for online learning materials and the increased demand for medical professionals during the COVID-19 pandemic has made this approach more relevant. The pediatric anesthesiology fellowship curriculum at one institution transitioned from a traditional classroom model to a flipped classroom approach to optimize the educational experience and support learner well-being.

Approach

Utilizing the American Board of Anesthesiology (ABA) and Accreditation Council for Graduate Medical Education (ACGME) content outlines, a novel, comprehensive curriculum was developed focusing on core clinical material and board review with the goal of providing structured learning while alleviating the burden of completing board review independently to improve work-life balance and reduce the potential for burnout.

Evaluation

Graduates of the program from 2017 to 2021 evaluated the flipped classroom structure and its perceived impact on their educational outcomes, wellness, and career development via a one-time, voluntary survey. Results indicated improved participation rates and educational benefits with the flipped classroom structure compared to the traditional structure. Fellows preferred the flipped classroom approach to the traditional lecture approach (72.2% vs. 27%), and they preferred custom-made prework material to traditional textbooks and articles. Fellows benefited from having more time for personal activities and other scholarly work, as evidenced by open-ended responses.

Implications

The flipped classroom structure was perceived as beneficial by pediatric anesthesiology fellows because of increased educational engagement, alleviation of academic burden, and improved wellness due to more time for non-academic activities. Further study is needed to optimize and correlate the new curriculum with objective educational outcomes.

## Introduction

The flipped classroom approach to instruction, which prompts learners to preview content independently prior to in-person instruction so that in-person time can be spent more deeply and actively engaging with the content expert [1], is an increasingly common alternative to traditional lecture or didactic methods of instruction, in which knowledge content-delivery typically occurs in-person with the instructor and time spent more deeply engaging afterward can vary widely by learner [1]. Unlike a traditional, instructor-centered classroom setting, the flipped classroom approach creates a learner-centered environment that fosters discussion and responds to learners’ needs. Generally, the flipped classroom approach provides learners with “prework” materials, such as pre-recorded lectures, textbook chapters, articles, and case studies, for review prior to a synchronous educational session in which all learners actively participate and engage with the content with the support of the instructor. The in-person session implements active learning strategies by addressing learners’ questions, providing opportunities for discussion, and encouraging deeper thinking around key concepts [1].

The academic benefits of the flipped classroom approach have been widely reviewed in undergraduate and graduate medical education [1-3]. Generally, the extant literature suggests a preference for the flipped classroom approach over other methods [1,2,4,5]. Learners report a better utilization of their time, while faculty find that this approach supports lifelong learning and self-guided inquiry skills [4-7]. However, other studies report negligible benefits of the flipped classroom approach [1]. This may be explained by the fact that efficacy is highly dependent upon individual learner engagement with the prework [6,8]. Students are frustrated by inadequate time to prepare, too much prework, or a perceived lack of structure for synchronous sessions [5,7,8]. Faculty also report barriers, including insufficient student preparation and the burden of providing quality prework [5].

Therefore, it is important to review learner perceptions of the flipped classroom approach, especially in the field of graduate medical education where work-life balance is a major focus of well-being. Burnout remains a critical concern in medicine, particularly during training when rates are highest [9]. Significant sources of burnout include negative workplace environments, heavy clinical demands, and difficulty in balancing academic responsibilities with other duties [10,11].

The flipped classroom approach to fellow curriculum provides one avenue to alleviate burnout from traditional demands of didactic time. Aligning curriculum choices with adult learning theory, as the flipped classroom approach does, supports trainees in self-directing and managing their academic needs [12]. In fact, literature on curriculum reform already reflects this priority of reducing academic burden in residency [13,14]. However, a focused curriculum reform utilizing the flipped classroom approach has not yet been published within a hospital-based fellowship program. Thus, we set out to better understand adult learners’ perceptions of the flipped classroom approach in a pediatric anesthesiology fellowship program.

## Materials and methods

At our institution, the pediatric anesthesiology fellowship program had utilized the traditional classroom model for years to teach the basic pediatric curriculum. However, the introduction of a new certification exam for pediatric anesthesiology by the American Board of Anesthesiology (ABA) in 2013 provided an impetus for creating additional material and developing a more effective approach to teaching it. A flipped classroom approach has been shown to be of benefit in residency education, but no such curriculum had been published for an anesthesiology fellowship in which more advanced learning material was being incorporated. Thus, a new series was developed to create a comprehensive curriculum (Pediatric Anesthesiology Comprehensive Review Series, PACRS) that teaches both the core clinical material and provides ongoing board review throughout the academic year. In this new approach, a series of topics was chosen based on the ABA content and the Accreditation Council for Graduate Medical Education (ACGME) outline.

For each topic, prework consisted of a related textbook chapter and a short informational podcast and/or PowerPoint presentation, which was shared via e-mail communication and a shared online repository. This was followed by an in-person session with a faculty member and/or a problem-based learning discussion (PBLD) to review the material. The goal was to provide structured learning and to alleviate the burden of completing board reviews independently to improve work-life balance and reduce the potential for burnout.

We investigated the impact of the flipped classroom approach on fellow satisfaction with respect to their educational program, academic progress, career development, and well-being, as well as their completion of and engagement with the prework. Our research questions were: (1) Did pediatric anesthesiology fellows participate and engage with flipped classroom resources and materials? (2) What were the barriers to participation, and what aided engagement with curriculum materials and structure? (3) What impact did the flipped classroom structure have on the fellows' perceived education outcomes and perceived wellness/work-life balance? (4) How satisfied were fellows with the curriculum and education materials provided by this new structure?

The study (IRB202912965) was qualified as exempt research by the Institutional Review Board. Our participant population consisted of pediatric anesthesiology fellowship program graduates from the years 2017 to 2021. Participation was voluntary, and no identifiable data were collected, aside from the year of graduation from the fellowship. Exclusion criteria included graduates who are either current faculty members or co-authors of this study. A one-time, voluntary survey was sent to 28 participants who met inclusion criteria, and responses were automatically uploaded into REDCap (Vanderbilt University, Nashville, TN) on a secure server for data analysis purposes. A total of 14 questions were included in the survey (Table 1).

**Table 1:** Survey items

Key survey questions
Did you watch pre-recorded lectures and/or access the written materials (PowerPoints/slides) when offered in advance of live morning lectures?
Did you read provided reference material (textbooks, articles) when offered in advance of live morning lectures?
How much time, in an average week, did you spend engaging with pre-work (watching pre-recorded lectures, reviewing PowerPoints, reading written materials, etc.) for lectures?
What were the limiting factors (if any) to engaging with prework and/or interactive morning sessions? (Open-ended)
What factors (if any) aided you in engaging with prework and/or interactive morning sessions? (Open-ended)
How beneficial did you find the provided pre-work (pre-recorded lectures, PowerPoints, etc.), on a scale from 1 to 5?
How beneficial did you find the interactive morning sessions, on a scale from 1 to 5?
How beneficial did you find the combination of the pre-work and interactive morning sessions?
Given the choice between traditional didactics structures (live lecture only) and the flipped classroom approach (pre-work paired with interactive live sessions), which do you prefer?
Did having an organized, formal curriculum that utilized the flipped classroom approach allow you to use time during fellowship for alternate activities?
If yes, what other types of activities were you able to dedicate time to that you otherwise may have spent studying pediatric anesthesia material?

Participation survey questions asked respondents if they participated in assigned prework and how much time they spent on prework. This section also solicited open responses on any factors aiding or limiting engagement with prework and synchronous sessions. Satisfaction questions were based on a five-point Likert scale and asked respondents how beneficial the prework was, how beneficial the morning session was, their preference for educational approach, and wellness-related questions. We also solicited any other comments related to the curriculum via open-response questions.

## Results

A total of 11 individuals completed the survey (39% full response rate). Responses included representative graduates from each surveyed fellowship class. Descriptive statistics, such as the percentage of responses, were calculated for questions regarding engagement, preference of educational approach, and wellness. Open-response answers were grouped and analyzed thematically to add context to the quantitative data.

Engagement

The average fellow participation in prework activities was 77%. Fellows were more likely to engage in customized prework material (90.9%), such as narrated PowerPoint slides, than with textbook chapters or articles (63.6%). Most respondents found weekly prework required one to two hours of commitment.

Perceived educational outcomes

**Figure 1:**
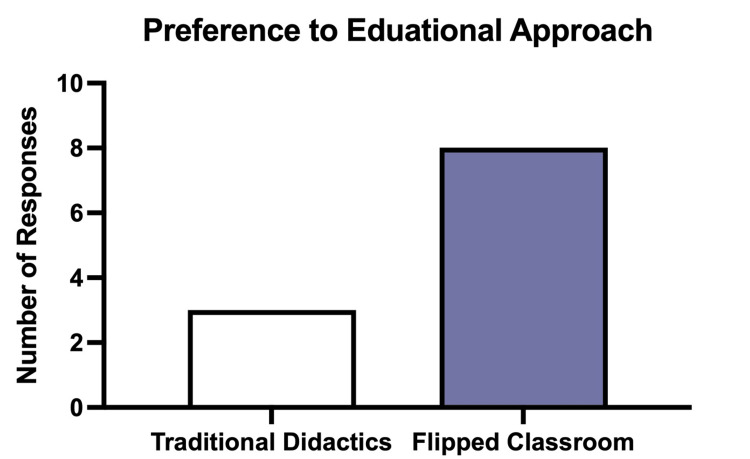
Respondent preference to educational approach: traditional didactics vs. flipped classroom

**Figure 2:**
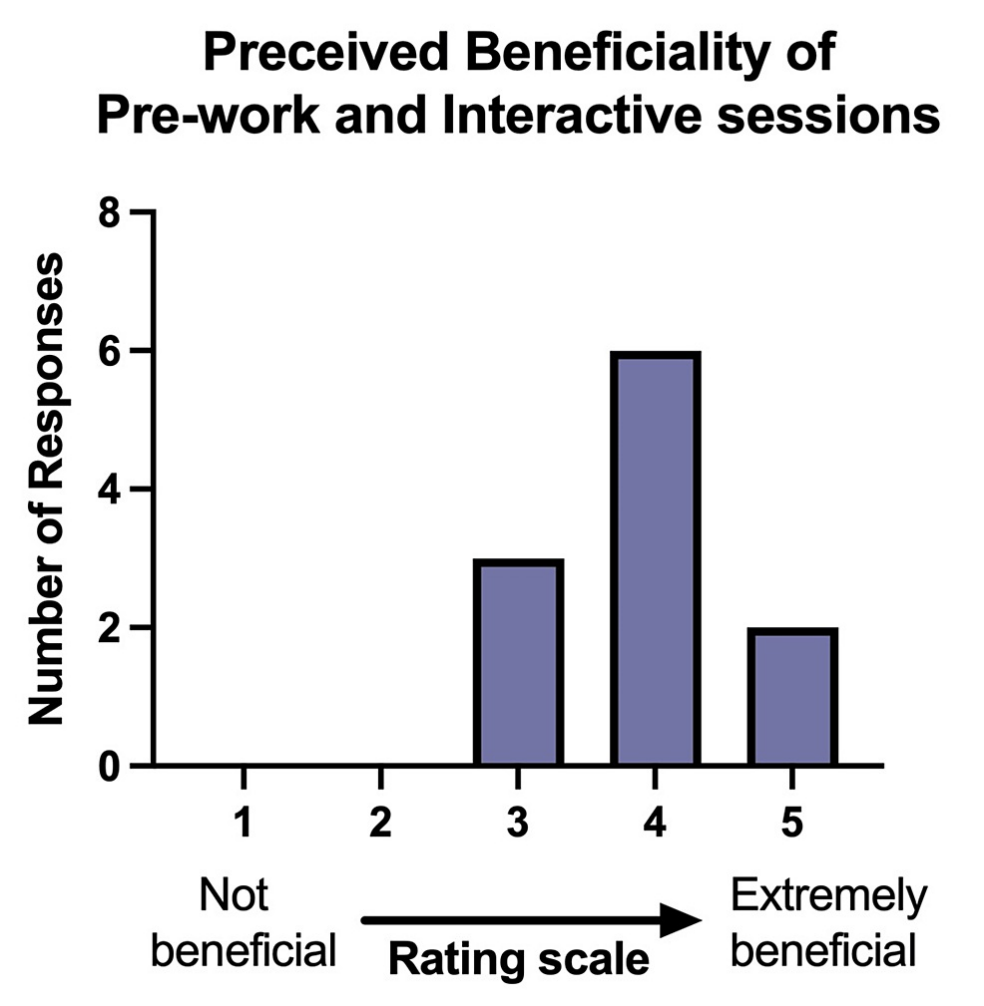
Respondent perceived beneficiality of the combination of prework interactive sessions

The majority of respondents overall (72.7%) preferred the flipped classroom approach when compared to a traditional classroom format (Figure 1). The perceived benefit of prework and interactive sessions was reported on a five-point scale, with one representing “not beneficial” and five representing “extremely beneficial.” Interactive synchronous sessions received a higher average benefit score than prework (4.27 vs. 3.55). Most fellows scored the benefits of the flipped classroom modality above three, with a median score of four (Figure 2).

Wellness

**Figure 3:**
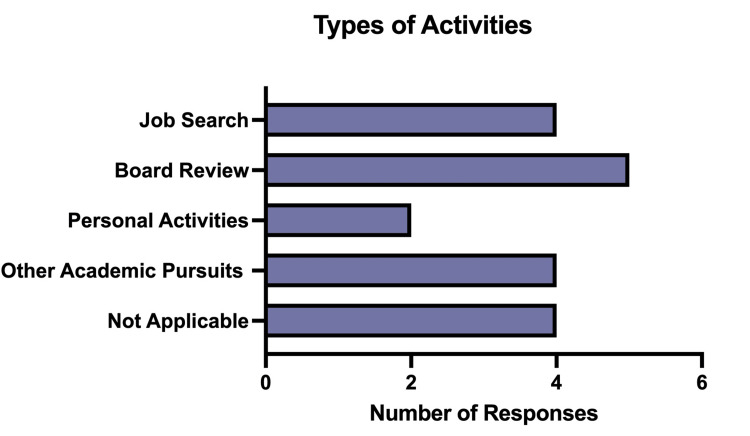
Types of activities respondents participated in due to increased free time

Approximately one-third of respondents found more time for alternate activities as a result of the flipped classroom approach, though it is unclear from our data how often respondents engaged in post-session learning activities with the traditional structure. Examples of alternate activities include job searching, personal activities, and board review (Figure 3).

Open-response narrative feedback

We offered participants the option to provide narrative comments related to the survey questions. Members of the research team independently reviewed these responses, and as a team, we identified three categories the comments fit within: positive aspects of the curriculum, barriers to curriculum use, and opportunities for improvement (Table 2).

**Table 2:** Exemplar narrative comments from open-response survey items

Group	Representative quotes
Positive elements	(1) I found reading the night before gave me a good knowledge base to feel confident to engage with the lecturer. (2) This curriculum helped me understand the topics better. It made me motivated to read up on the topic beforehand. (3) [The curriculum structure] helped prepare me for the boards. (4) It [the curriculum] definitely felt organized and well thought out, which results in an ideal learning environment.
Barriers to use	(1) Length of workday caus[ed] want of rest after the workday ends. (2) Reading to prepare for specific cases as well as board preparation [was challenging]. (3) The cardiac rotation or early morning cases made it hard to participate at times.
Opportunities for improvement	(1) Slides/handouts were easier to review than video or audio lectures, in terms of being able to take notes without having to pause the video. (2) Lectures felt disjointed, likely due to differing lecture styles and tactics. A progressive curriculum with limited faculty changeover might be helpful. (3) I only sometimes read the full [textbook] chapters, as time for reading the chapters was sometimes limiting. When there were specific resources, I would prioritize reading those and reviewing those. (4) Getting material on the weekend helped since I could study then.

In summary, fellows did engage in prework with a preference for custom material and an average time commitment of two hours or less. The flipped classroom model was generally preferred to a standard didactic format.

## Discussion

The flipped classroom approach was adopted to deliver streamlined, practical content and optimize the use of adult learning theory [1]. High participation from our sample in engaging with prework materials and satisfaction rates indicated reduced academic burden and stress for fellows. Specifically, fellows found the curriculum beneficial for board preparation and felt their weekly time investment in the structured curriculum was productive and practical, with over half of the survey respondents indicating they spent two hours or less on prework. This model also served as an essential improvement for a multi-site program; both prework and weekly synchronous sessions could be virtually accessed by individuals regardless of their location. Likewise, this model avoided COVID-19-related interruptions to in-person and traditional models of learning and remains adaptable for future use.

As Figure 3 highlights, we are especially interested in the potential this educational model offers in allowing fellows more time to devote to activities they determine are meaningful and important. The flipped classroom approach aligns with key principles of adult learning (Table 3), which supports and allows for self-management of learners’ academic needs [12] as several of our respondents indicated. Allowing adult learners to utilize time how they best see fit has the potential to decrease burnout, as control over time and schedule are key factors in the mitigation of feelings of burnout. Our fellows used their time to prepare for board exams, make progress in their job searches, and additional academic/personal pursuits.

**Table 3:** Key adult learning principles as reflected by elements of the flipped classroom curriculum in the pediatric anesthesiology fellowship program ACGME: Accreditation Council for Graduate Medical Education. Adapted from [12]. Knowles MS: The Modern Practice of Adult Education: Andragogy Versus Pedagogy. New York Association Press, New York, NY; 1970.

Adult learning principles [12]	Applications in curriculum
Autonomous and self-directed	Early access to prework permits autonomy in scheduling when and how tasks are completed
Bring prior knowledge and experiences to learning	Option to fast-forward or skip over familiar content to maximize time spent on new or unfamiliar content
Goal-oriented	Aligned to ACGME milestones and fellowship graduation components
Relevancy-oriented	Knowledge intended to be immediately applicable and useful in clinical practice
Practical	Allows learners to prioritize content as needed and relevant and optimizes time for other academic pursuits
Need to be shown respect	Implicit trust that learners will complete prework and respectful of learners’ time, with synchronous in-person sessions maximized for deeper learning

Study limitations include a small number of participants and only one institution. Additionally, the time between answering the survey and graduating, as well as the specific set of curricula resources, varied by participant. Another limitation, which warrants further exploration in future studies, is that we did not directly evaluate how much time fellows were spending on extra learning in the traditional classroom structure. This limits our ability to draw conclusions that the flipped classroom is more effective from a time-saving perspective. Lastly, the association of this curriculum with an objective outcome, such as board exam score, was difficult to discern and not specifically investigated. Future evaluation of which structure is more effective at improving learning and knowledge gain would be beneficial.

Despite these limitations in the study design, the narrative feedback described concrete areas for further improvement. Specifically, fellows found it difficult to complete the prework during more clinically demanding rotations. They also found the prework had variable quality and consistency, as it was produced by different faculty members. In addition, certain prework structures were more user-friendly than others. This feedback will be instrumental in improving this curriculum for future fellows.

## Conclusions

In summary, the adoption of a flipped classroom structure for the pediatric anesthesiology fellowship program was perceived as a success by graduates. Former fellows reported an improvement in participation, satisfaction, and educational outcomes with the flipped classroom structure compared to a traditional classroom model. Specific areas for development and barriers to use were highlighted by the narrative feedback, such as determining the most effective type of prework and refining strategies to balance academically and clinically demanding workloads.

Further investigation such as the impact of the flipped classroom curriculum in relation to board exam scores would provide an objective measure of the curriculum’s success. If studied in relation to topic-specific board exam results, this will also delineate the most effective content to integrate. Thus, regular assessment of the efficacy of this curriculum will enable iterative improvements that will support our fellows’ development into pediatric anesthesiologists.
